# Sexism, racism, and nationalism: Factors associated with the 2016 U.S. presidential election results?

**DOI:** 10.1371/journal.pone.0229432

**Published:** 2020-03-09

**Authors:** Natalie J. Shook, Holly N. Fitzgerald, Shelby T. Boggs, Cameron G. Ford, Patricia D. Hopkins, Nicole M. Silva

**Affiliations:** 1 West Virginia University, Morgantown, WV, United States of America; 2 School of Nursing, University of Connecticut, Storrs, CT, United States of America; Georgia State University, UNITED STATES

## Abstract

After the generally unexpected outcome of the 2016 U.S. presidential election, many explanations were proposed to account for the results. Three narratives that received a considerable amount of media attention were that sexist, racist, and/or nationalist attitudes influenced voting decisions. Some empirical work has supported each of these accounts. However, sexism, racism, and nationalism are interrelated, and most studies about the 2016 election have not examined these three factors in conjunction to determine the unique contribution of each. Thus, we investigated the extent to which each factor (assessed as sexism toward women, Modern Racism, and U.S. nationalism) was uniquely related to evaluations of Hillary Clinton and Donald Trump, voting intentions, and actual voting behavior. Participants completed online questionnaires before (*N* = 489) and after (*N* = 192) the 2016 U.S. election. More positive evaluation of Clinton and intentions to vote for Clinton were associated with lower levels of Modern Racism. More positive evaluation of Trump was associated with greater sexism toward women, Modern Racism, and U.S. nationalism. Intent to vote for Trump was associated with greater sexism toward women and Modern Racism. However, only Modern Racism significantly predicted voting behavior. Greater Modern Racism was associated with greater likelihood of voting for Trump and lower likelihood of voting for Clinton. When considered in conjunction, Modern Racism was the most consistent predictor across the different election outcome variables. Sexism toward women and U.S. nationalism were generally not significantly related to evaluations, intentions to vote, or voting behavior when accounting for Modern Racism. Thus, our data indicate that Modern Racism was correlated with vote choice in the 2016 election.

The 2016 U.S. presidential election was historic, not only because Hillary Clinton was the first female presidential candidate for a major political party, but also because of the generally surprising results. Going into the 2016 U.S. presidential election, most polls projected Hillary Clinton to win the election over Donald Trump [1]. Instead, Trump won the election with 304 Electoral College votes to Clinton’s 227 votes. Following the largely unanticipated outcome of the election, several narratives predominated the media as explanations for the election results. Some proposed that Clinton’s defeat was due to sexism toward women (e.g., [2,3]). Others argued that the election demonstrated underlying racist sentiments in the U.S. (e.g., [4,5]). A third explanation was that growing nationalistic attitudes in the U.S. contributed to Trump’s win (e.g., [6,7]). Although scholars have found evidence to support each of these accounts (e.g., [8,9,10]), most studies did not consider all three factors in conjunction. This is important as sexism toward women, racism, and nationalism are generally correlated with one another (e.g., [11]), so it is unclear to what extent each factor was uniquely related to the election outcome. Furthermore, many of the studies did not prospectively test the association between these factors and voting behavior. Thus, the goal of this study was to determine the extent to which sexism toward women, racism, and nationalism independently accounted for attitudes toward the presidential candidates and voting intentions pre-election, as well as predicted actual voting behavior.

## Sexism

Despite advances in gender equality in the workforce in the last few decades, gender gaps still remain in hiring, promotion, and salaries (e.g., [12,13]). For example, only 23.7% of the 2019 U.S. congress are women [14]. This inequality is in part due to gender stereotypes and sexist beliefs about women (e.g., [15]). In particular, gender inequality in leadership positions is related to match/mismatch in societal stereotypes regarding gender and leader roles (see [16], for a review). According to role congruity theory, female leaders are evaluated more negatively because there is inconsistency in the traits associated with their gender (e.g., dependent, emotional) and those of a leader (e.g., strength, rational; [17]). Male leaders, on the other hand, are evaluated more positively because they are perceived as possessing more leadership qualities, due to overlap in societal stereotypes. This bias applies to evaluations of presidential candidates. In an experimental study, a hypothetical female presidential candidate was evaluated more negatively than a hypothetical male presidential candidate with the exact same qualifications [18]. However, it should be noted that this study had a small sample size and thus low statistical power. In another study, actual female presidential candidates (i.e., Hillary Clinton, Elizabeth Dole) were similarly evaluated as less qualified than male candidates (i.e., John Edwards, Rudy Giuliani, and John McCain), and participants were more likely to vote for male candidates than female candidates [19].

Women who are perceived as more agentic when seeking a leadership position often face backlash in the form of hostile sexism [20]. According to ambivalent sexism theory, sexism toward women consists of two components: hostile and benevolent sexism [21,22]. Hostile sexism refers to more traditional prejudice and hostility toward women, and it is based on beliefs that women are threatening men’s position and power. Benevolent sexism includes attitudes of appreciation for women, but is based on beliefs that women are weaker than men and traditional gender roles should remain in society. As such, men should protect women. These seemingly contradictory aspects of sexism toward women help to maintain men’s greater status in society while simultaneously recognizing the necessity for men and women to have favorable relations for reproductive purposes. In order to maintain the status quo, women are overtly criticized and evaluated negatively when they do not prescribe to traditional gender roles (i.e., hostile sexism), and they are viewed positively as caring and in need of protection from men when in traditional gender roles (i.e., benevolent sexism; [23]). In general, Hillary Clinton is viewed as less stereotypically feminine, and greater hostile sexism toward women has previously been associated with lower likelihood of voting for Hillary Clinton [24].

With regard to the 2016 election, several studies have found that greater endorsement of sexist beliefs about women was related to voting for Trump (e.g., [25,26]). In particular, Bock et al. [8] found that undergraduate students who endorsed greater hostile sexism toward women (post-election) were more likely to have voted for Trump instead of Clinton. In nationally representative samples, greater hostile sexism toward women (pre-election) predicted voting for Trump instead of Clinton [27,9]. Thus, Clinton’s loss may have been due to gender stereotypes and sexist beliefs about women that deemed her as less competent than a male candidate. Given negative evaluations of women in nontraditional roles (e.g., Clinton running for president), sexism toward women, particularly hostile sexism, might have played a role in voting behavior during the 2016 presidential election. Clinton’s campaign as the first female president may have challenged societal stereotypes and indicated a push toward further equality and against traditional sexist attitudes toward women.

## Racism

Some political analysts have argued that the results of the 2016 U.S. presidential election demonstrated the continued presence of racism in the U.S. During the Obama presidency and with the growing diversity of the U.S. population, a portion of White Americans may have felt that their status as the majority was being threatened [28,5,29,30]. Feeling that they were not being represented and that their needs were not being met may have fed into racist sentiments. Trump’s presidential campaign may have appealed to this group by giving voice to their concerns about their place in the country. For example, Trump made several comments during the campaign that were deemed racist (e.g., suggesting that some Mexican immigrants are rapists and criminals) and some of his proposed policies were viewed as instilling racist attitudes (e.g., advocating for a Muslim-specific travel ban; [31,32]). With regard specifically to anti-Black racism, several individuals, including Clinton, alleged that Trump had previously discriminated against Black renters [33–35]. Additionally, Trump promoted a conspiracy theory that former President Barack Obama was not a U.S. citizen–a theory some have deemed racist [34]. These issues were raised during the 2016 campaign cycle [36–35]. Experimental work has demonstrated that presenting information about changing U.S. demographics (i.e., increasing minority group representation) to White individuals who strongly identify with their racial group induces group status threat and increases support for Trump [37]. Thus, Trump’s perceived endorsement of racism and dissatisfaction with Obama’s presidency by status threatened White Americans may have increased support for Trump’s candidacy.

Some research has examined the role of racism, particularly Modern Racism, in politics. Since the Equal Rights Movement in the U.S., there has been a significant shift in social norms regarding the open expression of racism (e.g., [38]). In contemporary times, traditional, overt forms of racism and discrimination are generally not socially acceptable. As such, racism has become more subtle and covert, and old-fashioned measures of racism are generally not valid (i.e., most respondents provide socially desirable answers). Modern Racism is a more subtle form of prejudice that is conceptualized as anti-Black feelings and beliefs that are expressed in such a way that they can easily be concealed or explained away [38,39,40]. That is, rather than endorsing overt forms of discrimination and prejudice (e.g., segregation), individuals high in Modern Racism are more likely to support policies that indirectly disadvantage African Americans (e.g., ending affirmative action). Dwyer and colleagues [41] proposed that Modern Racism played a significant role in the 2008 U.S. presidential election. They found that greater Modern Racism was associated with more negative evaluations of Barack Obama *and* more positive evaluations of Sarah Palin, the Republican vice-presidential candidate. Interestingly, ambivalent sexism toward women did not predict evaluations of Sarah Palin or Barack Obama, when examined in conjunction with Modern Racism. Thus, Modern Racism is related to political attitudes [42–44] and may play a unique role above and beyond other forms of bias.

With regard to the 2016 election, a few studies have found that greater endorsement of racism or less concern about racism were related to voting for Trump instead of Clinton (e.g., [26,9]). Given suggestions about the status threat of some White Americans and Trump’s campaign rhetoric, Modern Racism may have contributed to the results of the 2016 presidential election. Those higher in Modern Racism may have been more inclined to endorse Trump’s views, whereas those lower in Modern Racism may have preferred Clinton’s message of unity–“Stronger Together.”

## Nationalism

An additional narrative that received media attention was the idea that growing U.S. nationalism contributed to Trump winning the presidential election. Nationalism refers to the idea that one’s country is superior to other nations [45]. A related, but distinct, construct is patriotism, or the degree to which an individual loves and takes pride in their country [45]. Although both nationalism and patriotism are associated with loyalty and love for one’s nation, nationalism (unlike patriotism) is associated with hostility toward other nations [46]. Indeed, nationalism is positively correlated with negative attitudes toward immigrants [47], social dominance orientation [48], military action [49], and prejudicial attitudes [11]. Trump’s stance on stricter immigration laws, a stronger military, and reduced globalization during the campaign may have appealed to more nationalistic voters.

Within the U.S., some research has examined civic nationalism, or nationalism based on the ideology of what it means to be American (e.g., freedom, equality, democracy; [50]). Several studies have investigated how attitudes toward symbols that represent American ideology (e.g., the American flag represents the freedom of America; [51]) are associated with nationalism. For example, Kemmelmeier and Winter [52] tested the effect of exposure to the American flag on feelings of nationalism. Participants in a room with an American flag reported higher levels of nationalism than those who had no American flag in the testing room. Patriotism did not differ between the two groups. Some scholars have argued that the use of language and symbols that increase nationalistic attitudes helped build support for the Iraq war after 9/11 and that nationalism affected foreign policy [53,51]. There is also evidence that nationalism differs along political party lines. Republicans/Conservatives tend to more strongly endorse nationalistic attitudes than Democrats/Liberals [45,54–57].

Very little research has examined the potential role of nationalism in the 2016 election. Whitehead et al. [10] examined Christian nationalism and found that those higher in Christian nationalism were more likely to have voted for Trump. To date, no studies have examined U.S. nationalism. With Trump’s slogan of “Make America Great Again!” and his stance of “America First,” the Trump campaign may have tapped into U.S. nationalist ideals. Conversely, Clinton’s more internationalist (i.e., appreciation for other nations) stance may have appealed to those lower in nationalism. Thus, support for Trump may have stemmed from pride in the U.S. over other nations.

## Current study

The results of the 2016 U.S. presidential election were surprising to many. Consequently, several reasons for the election outcome have been posed. The purpose of this study was to examine the extent to which sexism toward women, Modern Racism, and U.S. nationalism independently contributed to the election results. As part of a larger research project, a national sample of U.S. citizens completed measures of hostile sexism toward women, benevolent sexism toward women, Modern Racism, and U.S. nationalism before the 2016 presidential election. They also evaluated the two primary candidates (Clinton and Trump) and reported their voting intentions. After the election, participants reported for whom they voted. Thus, we examined the extent to which sexism toward women, Modern Racism, and U.S. nationalism each uniquely accounted for evaluations of Clinton and Trump, voting intentions, and voting behavior.

It was expected that greater sexism toward women, particularly hostile sexism toward women, would be associated with more positive evaluation of Trump, less positive evaluation of Clinton, greater intention and likelihood of voting for Trump, and lesser intention and likelihood of voting for Clinton. Greater Modern Racism was expected be associated with more positive evaluation of Trump, less positive evaluation of Clinton, greater intention and likelihood of voting for Trump, and lesser intention and likelihood of voting for Clinton. Greater U.S. nationalism was expected to be associated with more positive evaluation of Trump, less positive evaluation of Clinton, greater intention and likelihood of voting for Trump, and lesser intention and likelihood of voting for Clinton.

It was unclear whether one factor would be a stronger predictor than the others, as sexism toward women, Modern Racism, and U.S. nationalism have generally not been examined in conjunction. When they have, the results have been mixed. For example, Dwyer et al.’s [41] work suggested that Modern Racism may be a stronger predictor than sexism toward women, but this may have been due to the 2008 U.S. presidential election including the first African American candidate. Three studies assessed both sexism toward women and racism predicting 2016 election voting intentions or behavior [26,9]. Both factors were significant predictors, and they were generally equivalent in strength. Whitehead et al. [10] considered Christian nationalism, racism, and sexism toward women. Christian nationalism was a significant predictor of voting behavior, whereas racism and sexism toward women were not significant predictors. Thus, we did not have specific hypotheses regarding comparisons between the three factors.

## Method

West Virginia University Institutional Review Board approved this research (protocol # 1311139985). All studies were conducted online, so participants provided electronic consent.

## Participants

A total of 489 participants were recruited through Amazon’s Mechanical Turk (MTurk). MTurk started as a crowdsourcing tool for small tasks, but is now widely used for rapid data collection by recruiting larger and diverse research participant pools at relatively inexpensive costs compared to traditional data collection methods [58,59,60]. Compared to community samples, data collected from MTurk are at least as valid and reliable as other methods [58–61]. Furthermore, the demographics of participants from MTurk do not differ drastically from participants recruited through other survey platforms [62]. However, it must also be noted that surveys listed on MTurk may receive low-quality responses from automated programs [63]. Although data were collected prior to this issue becoming a large concern, we extensively screened data to ensure exclusion of duplicate responses.

Participants were recruited for a larger study regarding individual differences, political attitudes, and behavior, which required a minimum sample size of 450 based on *a priori* power analysis (α = .05, power = .80) for detecting a small effect size. The only inclusion criteria were that participants had to be 18 years or older and U.S. citizens. For the current research questions, the sample size met several rules-of-thumb for minimum sample size required for structural equation modeling (see [64], for discussion). The full sample consisted of 55.5% women and was aged 19 to 81 years (*M*_age_ = 37.10 years, *SD* = 11.73). The racial/ethnic breakdown of the sample was White (77.5%), African American/Black (10.4%), Hispanic/Latino (5.9%), Asian (3.6%), Native American/Pacific Islander (0.7%), and ‘Other’ (1.9%). With regard to political party affiliation, 37.6% identified as Democrat, 28.6% identified as Republican, 27.4% identified as Independent, 3.8% identified as Libertarian, and 2.6% identified as ‘Other.’ Participants came from 42 of the 50 U.S. states. Sixty-seven participants did not provide demographic information.

### Measures

#### Voting intentions and behavior

Before the 2016 U.S. presidential election, participants were asked if they intended to vote in the election (yes or no). For those who intended to vote, they were asked for whom they would vote (Hillary Clinton, Gary Johnson, Jill Stein, Donald Trump, or Other). After the 2016 U.S. presidential election, participants were asked if they voted in the election (yes or no). For those who voted, they were then asked to indicate for whom they voted (Hillary Clinton, Gary Johnson, Jill Stein, Donald Trump, or Other).

#### Presidential candidate evaluations

Participants were asked to evaluate Donald Trump and Hillary Clinton on several dimensions. Participants indicated their general attitude toward each candidate on a scale from 0 (*cold or unfavorable*) to 100 (*warm or favorable*). Participants also indicated the extent to which they found each candidate likeable, trustworthy, knowledgeable, and competent on a scale from 1 (*not at all*) to 5 (*very*). The presentation of these items was randomized for each participant. All of the items evaluating Donald Trump (*r*s: .80 - .89; α = .96) and Hillary Clinton (*r*s: .56 - .88; α = .93) were strongly correlated. For descriptive purposes, a composite evaluation score for each candidate was created by standardizing each item and averaging them together. Higher values indicate a more positive evaluation.

#### Modern racism scale [38]

This questionnaire consists of seven items. Participants rated their agreement with each item (e.g., “Discrimination against Blacks is no longer a problem in the U.S.”) on a scale from -2 (*disagree strongly*) to 2 (*agree strongly*). The construct validity of the modern racism scale is well-established (see [65], for a review). For example, the Modern Racism Scale is positively correlated with measures of old-fashioned racism, implicit measures of prejudice toward Black individuals, opposition to policies that would benefit Black individuals (e.g., busing programs), and reluctance to hire Black applicants or vote for Black candidates (e.g., [66,38]). Mean scores were calculated with higher values indicating greater prejudice towards African Americans (α = .93).

Concerns have been raised that some measures of Modern Racism, particularly the Symbolic Racism Scale, confound racist beliefs with conservative values [67,68]. These critiques have not been raised with regard to the measure we used. Indeed, McConahay [38] developed the Modern Racism Scale to eliminate potential confounds and directly assess negative attitudes toward Black individuals. However, there is one item (“Over the past few years, Blacks have gotten more economically than they deserve”) that could be argued to potentially tap into economic conservative values. As such, we re-ran all primary analyses excluding this one economic item from the Modern Racism Scale (α = .91). The pattern of results did not differ.

#### Ambivalent sexism inventory [21]

This questionnaire consists of 22 items total and has two subscales: hostile (e.g., “women are too easily offended”) and benevolent (e.g., “many women have a purity quality few men possess”) sexism toward women. Each subscale consists of 11 items. Participants indicated the extent to which they agreed with each statement on a scale from 0 (*disagree strongly*) to 5 (*agree strongly*). Mean scores were calculated for each subscale with higher values indicating greater benevolent (α = .89) and hostile (α = .92) sexism toward women.

#### Patriotism/Nationalism Questionnaire [45]

The nationalism subscale of the Patriotism/Nationalism Questionnaire was used to assess U.S. nationalism. Participants indicated the extent to which they agreed or disagreed with eight items (e.g., “Other countries should try to make their government as much like ours as possible”) on a scale from 1 (*strongly disagree*) to 5 (*strongly agree*). Mean scores were calculated with higher values indicating greater nationalism (α = .88).

#### Demographics

Participants were asked to provide their age, gender, race/ethnicity, home state, and political party affiliation (i.e., Democrat, Republican, Libertarian, Independent, or Other).

#### Procedure

Participants were recruited through MTurk. They registered for a two-part online study about political beliefs and voting. The first part of the study occurred between October 20 and November 7, 2016. Informed consent was obtained electronically from all individual participants included in the study. After providing electronic consent, participants completed an online survey. First, they were asked about their voting intentions. Next, they were asked to evaluate Hillary Clinton and Donald Trump. Then, they were presented with a series of questionnaires in a random order that included the Modern Racism Scale, the Ambivalent Sexism Inventory, and the Patriotism/Nationalism Questionnaire. Finally, participants completed the demographics questions. Upon completion of the survey, participants received $1.00.

The second part of the study occurred between November 9 (the day after the 2016 U.S. presidential election) and January 11, 2017. After the 2016 U.S. presidential election, participants received notification that they could complete the second part of the study and were provided with a secure link to an online survey administered through SurveyMonkey. The survey consisted of a number of questionnaires about social attitudes, individual differences, and emotions for the larger project. Of relevance for the current study, participants were asked about their voting behavior during the presidential election. Upon completion of the survey, participants received $1.50.

### Analytic strategy

Structural equation modeling (SEM) was utilized to assess the extent to which sexism toward women, Modern Racism, and U.S. nationalism were uniquely associated with evaluations of Hillary Clinton and Donald Trump, voting intentions, and voting behavior. SEM was chosen as it eliminates concerns of multicollinearity [69,70]. Item-level indicators were used to specify latent variables representing Modern Racism, benevolent sexism toward women, hostile sexism toward women, U.S. nationalism, and evaluations of Clinton and Trump. Some participants did not fully complete the measures of Modern Racism (1.63%), sexism toward women (6.95%), U.S. nationalism (5.93%), or demographic information (13.70%). Little’s MCAR test was non-significant, indicating that the data appeared to be missing completely at random [71]. To address missing data, Full Information Maximum Likelihood estimation was performed to find maximum likelihood estimates for model parameters. Model fit was evaluated with standard metrics, and acceptable fit was indicated with χ2/*df* < 3.0, comparative fit index (CFI) > .90, and root mean square error of approximation (RMSEA) < .08 [72]. All analyses were performed in IBM SPSS Amos 23.

## Results

### Descriptive statistics

During the first part of the study, participants were asked whether they intended to vote in the 2016 U.S. presidential election. Out of 489 participants, 436 (89.7%) indicated that they intended to vote. Of those, 234 (51.7%) intended to vote for Hillary Clinton, 30 (6.6%) intended to vote for Gary Johnson, 17 (3.8%) intended to vote for Jill Stein, 148 (32.7%) intended to vote for Donald Trump, and 24 (5.3%) intended to vote for someone else. As the main research question concerned understanding the 2016 election outcome, only participants who intended to vote were included in the subsequent analyses. For the sample that intended to vote, 54.8% were women, and age ranged from 19 to 81 years (*M*_age_ = 37.46 years, *SD* = 11.77). The racial/ethnic breakdown of the sample was White (80.4%), African American/Black (9.8%), Hispanic/Latino (5.8%), Asian (3.2%), Native American/Pacific Islander (0.8%), and ‘Other’ (1.3%). With regard to political party affiliation, 39.4% identified as Democrat, 29.6% identified as Republican, 25.1% identified as Independent, 4.2% identified as Libertarian, and 2.6% identified as ‘Other.’ Compared to those who intended to vote, excluded participants (i.e., those who did not intend to vote) were less likely to be White (*p* < .001) and evaluated Trump and Clinton more positively (*p*s < .001). Excluded participants did not differ from those who intended to vote along any other demographic variables (*p*s > .53).

Means and standard deviations for all study variables are displayed in Table 1. To assess the simple associations among all study variables, bivariate correlations were estimated (see Table 2). As all participants were included in the SEM analyses, correlations are reported with cases excluded pairwise. The pattern of results did not differ if cases were excluded listwise or if maximum likelihood estimates were included for missing scores. Modern racism, hostile sexism toward women, benevolent sexism toward women, and U.S. nationalism were all significantly, positively correlated with one another. More positive evaluation of Trump and intent to vote for Trump were significantly associated with greater Modern Racism, hostile sexism toward women, benevolent sexism toward women, and U.S. nationalism. More positive evaluation of Clinton and intent to vote for Clinton were all significantly, inversely correlated with Modern Racism, hostile sexism toward women, benevolent sexism toward women, and U.S. nationalism. Also, evaluation of Clinton and intent to vote for Clinton were all significantly, inversely correlated with evaluation of Trump and intent to vote for Trump.

**Table 1:** Descriptive statistics for measures for participants who intended to vote at Time 1 for the full sample and split by political party.

	*Full Sample*			*Democrats*			*Republicans*			*Independent*			*Libertarian*			*Other*		
	*M*	*SD*	*N*	*M*	*SD*	*N*	*M*	*SD*	*N*	*M*	*SD*	*N*	*M*	*SD*	*N*	*M*	*SD*	*N*
Modern Racism	-0.75	1.05	428	-1.17	.88	149	-0.01	0.95	112	-.79	.99	95	-.87	1.15	16	-1.90	0.15	6
Hostile Sexism	1.87	1.12	406	1.34	1.05	149	2.48	0.90	112	2.12	1.07	95	1.52	1.06	16	1.30	1.32	6
Benevolent Sexism	2.19	1.03	406	2.02	1.01	149	2.59	0.99	112	2.14	1.01	95	1.77	.75	16	1.42	0.98	6
Nationalism	2.90	0.81	410	2.65	.82	148	3.40	0.69	112	2.76	.63	95	2.73	.64	16	1.92	0.86	6
Clinton Evaluation	0.13	0.90	435	.85	.57	149	-0.62	0.69	112	-.13	.71	95	-.15	.91	16	-0.20	0.74	6
Trump Evaluation	-0.04	0.94	435	-.60	.62	149	0.87	0.81	112	-.06	.85	95	-.32	.83	16	-0.59	0.36	6
Intend to Vote Clinton	0.52	0.50	435	.94	.24	149	0.12	0.32	112	.37	.48	95	.31	.48	16	0.17	0.41	6
Intend to Vote Trump	0.34	0.47	435	.03	.16	149	0.82	0.38	112	.36	.48	95	.19	.40	16	0.17	0.41	6

Intend to Vote/Voted Clinton coded 0 = other candidate, 1 = Clinton. Intend to Vote/Voted Trump coded 0 = other candidate, 1 = Trump.

**Table 2:** Zero-order correlations among measures for participants who intended to vote at Time 1.

	2	3	4	5	6	7	8
1. Modern Racism	.59**	.32**	.45**	-.43**	.58**	-.41**	.51**
2. Hostile Sexism		.40**	.49**	-.40**	.49**	-.37**	.40**
3. Benevolent Sexism			.55**	-.13**	.25**	-.15**	.14**
4. Nationalism				-.27**	.44**	-.24**	.33**
5. Clinton Evaluation					-.62**	.79**	-.68**
6. Trump Evaluation						-.66**	.81**
7. Intend to Vote Clinton							-.75**
8. Intend to Vote Trump							

Intend to Vote/Voted Clinton coded 0 = other candidate, 1 = Clinton. Intend to Vote/Voted Trump coded 0 = other candidate, 1 = Trump. Cases were excluded pairwise.

** *p* < .01

The relations between demographic variables and the primary study variables were also assessed. Women reported lower Modern Racism (*r* = -.20, *p* < .001), lower U.S. nationalism (*r* = -.11, *p* = .031), lower hostile sexism toward women (*r* = -.25, *p* < .001), lower benevolent sexism toward women (*r* = -.11, *p* = .038), more negative evaluation of Trump (*r* = -.17, *p* = .001), and lower intention to vote for Trump (*r* = -.11, *p* = .029) than men. Age was unrelated to any study variables. Racial/ethnic minority participants reported lower Modern Racism (*r* = -.12, *p* = .017), more positive evaluation of Clinton (*r* = .16, *p* = .002), more negative evaluation of Trump (*r* = -.16, *p* = .002), greater intent to vote for Clinton (*r* = .19, *p* < .001), and lower intent to vote for Trump (*r* = -.18, *p* = .001).

#### Evaluations of the candidates and voting intentions

**Fig 1:**
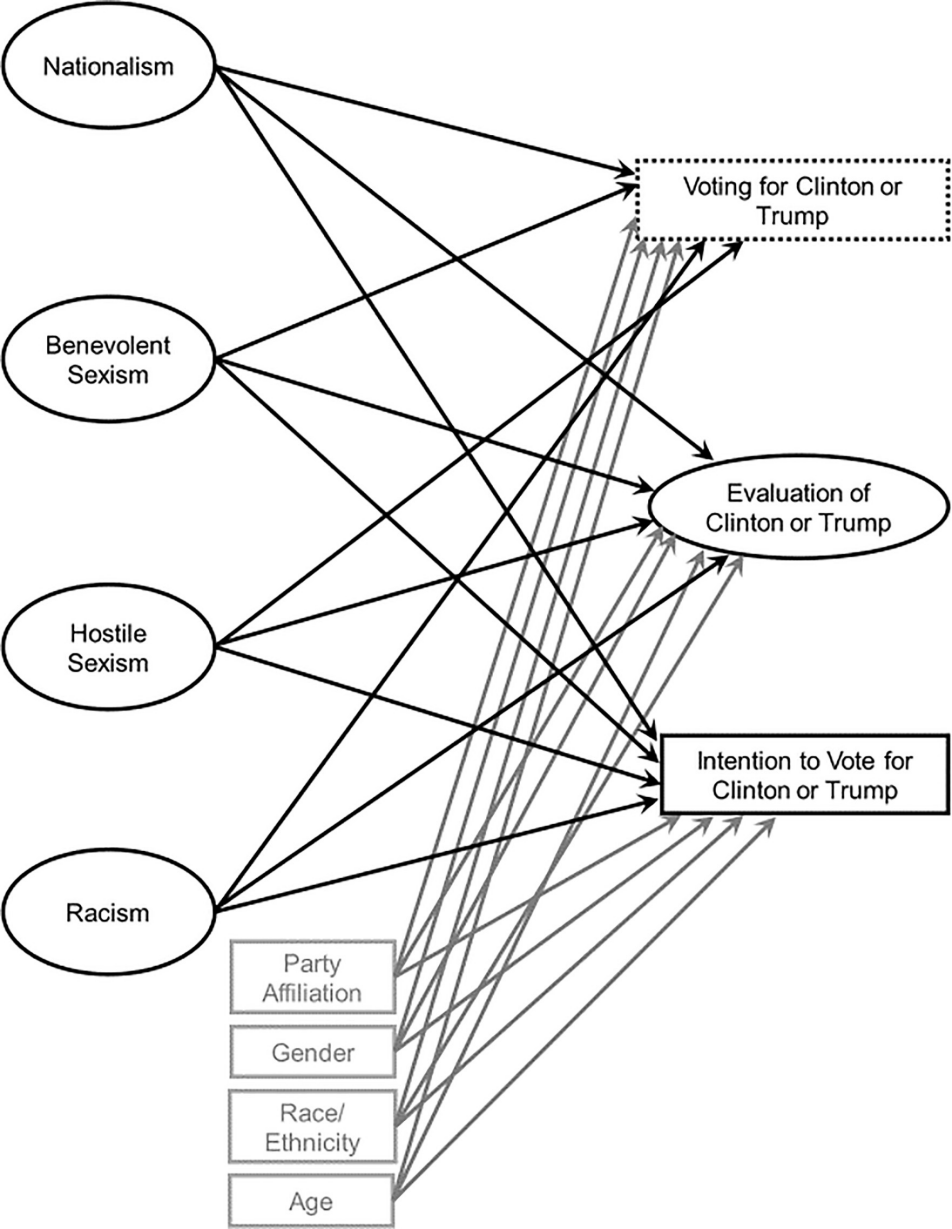
Conceptual figure depicting structural model testing the extent to which sexism toward women, modern racism, and nationalism were uniquely associated with evaluations of, intentions to vote for, and voting for Clinton or Trump. Separate models were tested for each candidate. Party affiliation, gender, race/ethnicity, and age were included as covariates.

Two structural models were estimated to determine the extent to which Modern Racism, sexism toward women, and U.S. nationalism were uniquely associated with evaluations of and intentions to vote for Clinton and Trump (see Fig 1 for a depiction of the models). One model focused on Clinton, and the other focused on Trump. Intent to vote for Clinton was coded such that 0 = other candidate and 1 = Clinton. Similarly, intent to vote for Trump was coded such that 0 = other candidate and 1 = Trump. Demographic variables (gender, age, race/ethnicity) were included as covariates, as each variable was related to several of the predictor or outcome variables. A dummy coded political party affiliation variable was included as a covariate, as party affiliation is a strong predictor of political attitudes and voting behavior (e.g., [73]). For analyses related to Clinton, party affiliation was coded (0 = other, 1 = Democrat). For analyses related to Trump, party affiliation was coded (0 = other, 1 = Republican).

With regard to Clinton, the model provided an acceptable fit to the data χ^2^/df = 1.803, RMSEA [95% Confidence Interval] = .043 [.040, .046], and CFI = .937. Table 3 displays the standardized estimates, the unstandardized estimates, and the standard errors for the model. Party affiliation was a significant predictor of evaluations of Clinton and intent to vote for Clinton. Democrats evaluated Clinton more positively and had greater intentions to vote for Clinton than those who affiliated with different political parties. Race was a significant predictor of intent to vote for Clinton, such that racial/ethnic minority participants had greater intentions to vote for Clinton than White participants. Modern racism was significantly negatively related to evaluation and intent to vote for Clinton. Individuals who more strongly endorsed modern racist beliefs evaluated Clinton more negatively and had lower intentions to vote for Clinton.

**Table 3:** Standardized estimates, unstandardized estimates, and standard errors for structural model predicting evaluation of Clinton and intent to vote for Clinton at Time 1.

	*Evaluation of Clinton*				*Intent to Vote for Clinton*			
Variable	*B*	*SE*	*β*	*p*	*B*	*SE*	*β*	*p*
Covariates								
Age	-0.01	0.01	-0.06	0.18	0.00	0.00	-0.03	0.43
Gender	-0.03	0.11	-0.01	0.80	-0.03	0.04	-0.03	0.39
Race	0.27	0.14	0.08	0.05	0.13	0.05	0.11	0.01
Party Affiliation	1.72	0.14	0.62	< .001	0.62	0.04	0.63	< .001
Main Predictors								
Modern Racism	-0.19	0.09	-0.14	0.02	-0.08	0.03	-0.17	0.003
Hostile Sexism	-0.13	0.08	-0.09	0.13	-0.02	0.03	-0.04	0.49
Benevolent Sexism	0.14	0.08	0.10	0.08	0.01	0.03	0.03	0.59
Nationalism	-0.11	0.09	-0.08	0.24	-0.02	0.03	-0.05	0.43

*N* = 436. Gender coded 0 = men, 1 = women. Race/Ethnicity coded 0 = white, 1 = non-white. Party Affiliation coded 0 = other, 1 = Democrat.

With regard to Trump, the model provided an acceptable fit to the data χ^2^/df = 1.766, RMSEA [95% Confidence Interval] = .042 [.039, .045], and CFI = .944. Table 4 displays the standardized estimates, the unstandardized estimates, and the standard errors for the model. Race/ethnicity and party affiliation were significant predictors of evaluations of Trump and intent to vote for Trump. Racial/ethnic minority participants evaluated Trump more negatively and reported lower intentions to vote for Trump than White participants. Participants who identified as Republican evaluated Trump more positively and had greater intentions to vote for Trump than those who identified with a different political party. Modern racism and hostile sexism toward women were each significantly positively related to evaluation of Trump and intention to vote for Trump. Individuals who more strongly endorsed Modern Racism and hostile sexism toward women evaluated Trump more positively and had greater intentions to vote for Trump. Benevolent sexism toward women was negatively related to intent to vote for Trump, such that individuals who more strongly endorsed benevolently sexist beliefs had lower intentions to vote for Trump. U.S. nationalism was significantly positively associated with evaluation of Trump. Those who endorsed greater U.S. nationalism evaluated Trump more positively.

**Table 4:** Standardized estimates, unstandardized estimates, and standard errors for structural model predicting evaluation of Trump and intent to vote for Trump at Time 1.

	*Evaluation of Trump*				*Intent to Vote for Trump*			
Variable	*B*	*SE*	*β*	*p*	*B*	*SE*	*β*	*p*
Covariates								
Age	0.00	0.01	0.01	0.78	0.00	0.00	0.02	0.71
Gender	-0.12	0.11	-0.04	0.29	-0.01	0.04	-0.01	0.87
Race	-0.37	0.14	-0.11	0.01	-0.10	0.05	-0.09	0.03
Party Affiliation	1.26	0.14	0.43	< .001	0.51	0.04	0.52	< .001
Main Predictors								
Modern Racism	0.37	0.08	0.27	< .001	0.11	0.03	0.24	< .001
Hostile Sexism	0.22	0.08	0.17	0.01	0.06	0.03	0.13	0.03
Benevolent Sexism	-0.12	0.08	-0.09	0.14	-0.07	0.03	-0.15	0.01
Nationalism	0.25	0.09	0.19	0.01	0.04	0.03	0.08	0.25

*N* = 436. Gender coded 0 = men, 1 = women. Race/Ethnicity coded 0 = white, 1 = non-white. Party Affiliation coded 0 = other, 1 = Republican.

#### Voting behavior

A total of 217 participants completed the second part of the study after the election. Participants who completed both parts of the study were compared to those who only completed the first part. Those who completed both parts evaluated Trump less positively, had lower intentions to vote for Trump, and were lower in hostile sexism toward women than those who only completed just the first part of the study (*p*s < .05). The two groups did not differ in age, gender, race, party affiliation, benevolent sexism toward women, Modern Racism, or U.S. nationalism (*p*s > .06).

Of the 217 participants, 192 participants voted in the election: 109 (56.5%) voted for Hillary Clinton, 9 (4.7%) voted for Gary Johnson, 8 (4.1%) voted for Jill Stein, 64 (33.2%) voted for Donald Trump, and 3 (1.6%) voted for someone else. As the main research question concerned understanding the 2016 election outcome, only participants who voted were included in the subsequent analyses. The sample was 56% women and aged 22 to 74 years (*M*_age_ = 38.66 years, *SD* = 11.76). The racial/ethnic breakdown of the sample was White (77.5%), African American/Black (11.0%), Hispanic/Latino (6.3%), Asian (2.1%), Native American/Pacific Islander (1%), and ‘Other’ (2.1%). With regard to political party affiliation, 42.7% identified as Democrat, 27.6% identified as Republican, 23.4% identified as Independent, 5.2% identified as Libertarian, and 1.0% identified as ‘Other.’ When compared to participants who voted, a greater percentage of excluded participants (i.e., those who did not vote) were non-White (*p* = .013), women (*p* < .001), and evaluated Clinton (*p* = .026) and Trump (*p* = .003) less positively. Excluded participants did not differ from those who voted along any other variables (*p*s > .057).

As the 192 participants who returned for session 2 and voted represented a subset of the initial sample, correlations between all study variables were rerun to determine whether the simple associations were different for this subgroup (see Table 5). Overall, the correlations between the pre-election (Time 1) variables replicated. With regard to voting, lower Modern Racism, hostile sexism toward women, benevolent sexism toward women, and U.S. nationalism were associated with higher likelihood of voting for Clinton. Greater Modern Racism, hostile sexism toward women, and U.S. nationalism were associated with greater likelihood of voting for Trump. More positive evaluation of Clinton and greater intention to vote for Clinton were associated with higher likelihood to voting for Clinton and lower likelihood to voting for Trump. More positive evaluation of Trump and greater intention to vote for Trump were associated with lower likelihood to voting for Clinton and higher likelihood to voting for Trump.

**Table 5:** Zero-order correlations among measures for participants who voted at Time 2.

	*M*	*SD*	N^a^	2	3	4	5	6	7	8	9	10
1. Modern Racism	-0.81	1.02	192	.58**	.28**	.43**	-.48**	.64**	-.44**	.53**	-.48**	.54**
2. Hostile Sexism	1.74	1.13	192		.40**	.43**	-.37**	.42**	-.31**	.31**	-.31**	.32**
3. Benevolent Sexism	2.15	1.02	192			.50**	-.16*	.25**	-.15*	.10	-.16*	.13
4. Nationalism	2.82	0.84	191				-.28**	.37**	-.22**	.31**	-.26**	.33**
5. Clinton Evaluation	0.22	0.91	192					-.64**	.79**	-.69**	.75**	-.69**
6. Trump Evaluation	-0.16	0.90	192						-.69**	.83**	-.67**	.79**
7. Intend to Vote Clinton	0.57	0.50	181							-.75**	.87**	-.74**
8. Intend to Vote Trump	0.29	0.46	181								-.73**	.87**
9. Voted Clinton	0.57	0.50	192									-.80**
10. Voted Trump	0.33	0.47	192									

Intend to Vote/Voted Clinton coded 0 = other candidate, 1 = Clinton. Intend to Vote/Voted Trump coded 0 = other candidate, 1 = Trump. ^a^ Cases were excluded pairwise.

* *p* < .05

** *p* < .01

Again, the relations between demographic variables and the primary study variables were assessed. Women reported lower Modern Racism (*r* = -.28, *p* < .001), lower hostile sexism toward women (*r* = -.31, *p* < .001), more negative evaluation of Trump (*r* = -.23, *p* = .001), and lower intention to vote for Trump (*r* = -.18, *p* = .018) than men. Younger age was associated with lower U.S. nationalism (*r* = -.16, *p* = .031). Racial/ethnic minority participants reported less Modern Racism (*r* = -.15, *p* = .043), more negative evaluation of Trump (*r* = -.18, *p* = .012), greater likelihood of voting for Clinton (*r* = .16, *p* = 0.24), and lower likelihood of voting for Trump (*r* = -.16, *p* = .028).

To determine the extent to which Modern Racism, sexism toward women, and U.S. nationalism were uniquely associated with voting for the primary candidates, two structural models were estimated–one for Clinton and one for Trump (see Fig 1). Voted for Clinton was coded such that 0 = other candidate and 1 = Clinton. Voted for Trump was coded such that 0 = other candidate and 1 = Trump. As participants who completed the second part of the study and voted may have been different than those who only completed the first part, evaluations and voting intentions were also included as outcomes in the model. Again, demographic variables and political party affiliation were entered as covariates.

With regard to Clinton, the model provided an acceptable fit to the data χ^2^/df = 1.559, RMSEA [95% Confidence Interval] = .054 [.049, .059], and CFI = .908. Table 6 displays the standardized estimates, the unstandardized estimates, and the standard errors for the model. Party affiliation significantly predicted voting for Clinton. Participants who identified as Democratic were more likely to have voted for Clinton than those who identified with a different political party. Modern racism also significantly predicted voting for Clinton. Individuals higher in Modern Racism were less likely to have voted for Clinton.

**Table 6:** Standardized estimates, unstandardized estimates, and standard errors for structural model predicting evaluation of Clinton, intent to vote for Clinton, and voting for Clinton at Time 2.

	*Evaluation of Clinton*				*Intent to Vote for Clinton*				*Voting for Clinton*			
Variable	*B*	*SE*	*β*	*p*	*B*	*SE*	*β*	*p*	*B*	*SE*	*β*	*p*
Covariates												
Age	-0.01	0.01	-0.06	0.34	0.00	0.00	-0.05	0.40	0.00	0.00	-0.05	0.37
Gender	-0.12	0.16	-0.04	0.46	-0.07	0.06	-0.07	0.24	-0.04	0.05	-0.04	0.42
Race	-0.06	0.19	-0.02	0.77	0.04	0.06	0.03	0.55	0.09	0.06	0.08	0.16
Party Affiliation	1.77	0.19	0.63	< .001	0.58	0.06	0.61	< .001	0.59	0.05	0.62	< .001
Main Predictors												
Modern Racism	-0.36	0.12	-0.26	0.00	-0.16	0.04	-0.34	< .001	-0.18	0.04	-0.37	< .001
Hostile Sexism	-0.05	0.11	-0.03	0.69	0.03	0.04	0.07	0.41	0.06	0.04	0.12	0.11
Benevolent Sexism	0.08	0.10	0.06	0.45	-0.01	0.03	-0.01	0.87	-0.01	0.03	-0.01	0.86
Nationalism	-0.01	0.11	-0.01	0.95	0.02	0.04	0.04	0.60	-0.01	0.03	-0.03	0.72

*N* = 192. Gender coded 0 = men, 1 = women. Race/Ethnicity coded 0 = white, 1 = non-white. Party Affiliation coded 0 = other, 1 = Democrat.

With regard to Trump, the model provided an acceptable fit to the data χ^2^/df = 1.465, RMSEA [95% Confidence Interval] = .049 [.044, .055], and CFI = .925. Table 7 displays the standardized estimates, the unstandardized estimates, and the standard errors for the model. Party affiliation was a significant predictor of voting for Trump. Participants who identified as Republican were more likely to have voted for Trump than those who identified with a different political party. Modern racism also significantly predicted voting for Trump. Individuals higher in Modern Racism were more likely to have voted for Trump.

**Table 7:** Standardized estimates, unstandardized estimates, and standard errors for structural model predicting evaluation of Trump, intent to vote for Trump, and voting for Trump at Time 2.

	*Evaluation of Trump*				*Intent to Vote for Trump*				*Voting for Trump*			
Variable	*B*	*SE*	*β*	*p*	*B*	*SE*	*β*	*p*	*B*	*SE*	*β*	*p*
Covariate												
Age	-0.01	0.01	-0.06	0.34	0.00	0.00	-0.04	0.53	0.00	0.00	0.00	0.94
Gender	-0.22	0.16	-0.08	0.18	-0.05	0.05	-0.06	0.38	-0.01	0.06	-0.01	0.86
Race	-0.34	0.19	-0.11	0.07	-0.05	0.06	-0.05	0.44	-0.08	0.07	-0.08	0.22
Party Affiliation	0.88	0.21	0.30	< .001	0.43	0.07	0.47	< .001	0.40	0.07	0.41	< .001
Main Predictors												
Modern Racism	0.75	0.13	0.56	< .001	0.15	0.04	0.36	< .001	0.16	0.04	0.37	< .001
Hostile Sexism	-0.11	0.11	-0.09	0.31	-0.02	0.04	-0.05	0.55	0.00	0.04	0.00	0.97
Benevolent Sexism	0.13	0.10	0.10	0.22	-0.03	0.03	-0.08	0.36	-0.03	0.04	-0.07	0.41
Nationalism	0.04	0.11	0.03	0.72	0.02	0.04	0.06	0.49	0.03	0.04	0.08	0.36

*N* = 192. Gender coded 0 = men, 1 = women. Race/Ethnicity coded 0 = white, 1 = non-white. Party Affiliation coded 0 = other, 1 = Republican.

## Discussion

The goal of the present study was to determine whether sexism toward women, Modern Racism, or U.S. nationalism may have played a role in the 2016 U.S. presidential election. Overall, the findings indicated that Modern Racism was most consistently related to evaluations of the presidential candidates, voting intentions, and voting behavior. Within the sample of individuals who intended to vote, more positive evaluation of Clinton and intentions to vote for Clinton were associated with lower levels of Modern Racism. More positive evaluation of Trump was associated with greater Modern Racism, hostile sexism toward women, and U.S. nationalism. Intent to vote for Trump was associated with greater Modern Racism and hostile sexism toward women, as well as less benevolent sexism toward women. However, only Modern Racism significantly predicted voting behavior. Greater Modern Racism was associated with greater likelihood of voting for Trump and lower likelihood of voting for Clinton. Importantly, these relations were found when controlling for demographic variables and party affiliation.

Of the three narratives proposed to explain the election outcome, the role of Modern Racism received the most consistent support in the current sample. Across the analyses related to both Clinton and Trump, Modern Racism was significantly associated with all outcome variables, independent of race, age, gender, party affiliation, sexism toward women, and U.S. nationalism. That is, Modern Racism was uniquely related to evaluations of and intentions to vote for Clinton and Trump. Moreover, Modern Racism prospectively predicted voting behavior. These findings align with previous research related to the 2008 U.S. presidential election, in which Modern Racism, but not ambivalent sexism toward women, was associated with evaluations of Barack Obama and Sarah Palin [41]. Our findings highlight the potential influence of Modern Racism in political attitudes and behavior, beyond simply whether one will vote for a Black versus a White candidate. Our findings align with work suggesting that status threat experienced by some majority group members may have influenced voting decisions [28,37].

An alternative explanation for the role of racism in the 2016 election has recently been proposed. According to Engelhardt [74], racial resentment did not increase on average within the Republican Party from 2012 to 2016; rather, racial resentment significantly decreased on average in the Democratic Party during that time. Thus, the Trump campaign may not have stoked racist attitudes. Instead, Clinton voters and the Democratic Party may have been more sensitive to or brought more attention to concerns of racism. Our data cannot address this possibility. Indeed, we found that participants who identified as Democrat endorsed lower levels of Modern Racism (*M* = -1.17, *SD* = .88) than participants who identified as Republican (*M* = -.01, *SD* = .95). However, we used a different measure of racism than the items used in Engelhardt [74]. As such, we cannot compare levels of racism across samples. It should also be noted that Engelhardt [74] used items from the Symbolic Racism Scale (e.g., “Irish, Italians, Jewish and many other minorities overcame prejudice and worked their way up. Blacks should do the same without any special favors”), which has been criticized for confounding racism and economic conservatism (e.g., [75–78]).

Sexism toward women, particularly hostile sexism toward women, was significantly associated with evaluations of and intentions to vote for Trump pre-election, independent of race, age, gender, party affiliation, Modern Racism, and U.S. nationalism. Traditional, hostile attitudes toward women were related to more positive evaluation of Trump and greater likelihood to vote for him. Although benevolent sexism toward women was associated with all of the outcome variables based on the zero-order correlations, benevolent sexism toward women did not remain significantly associated with the outcome variables when controlling for other factors, except for an inverse association with intent to vote for Trump, which was most likely a suppression effect [79]. Thus, sexist attitudes related to protecting women were not associated with the primary outcome variables. Rather, sexist attitudes related to women’s inferiority and the potential threat of women taking power away from men were significantly associated with more positive evaluations of the male candidate. Notably, however, hostile sexism toward women was correlated with evaluations of Clinton and intent to vote for Clinton, which aligns with role congruity theory and negative evaluations of nontraditional females, such as female leaders (e.g., [17,23]). However, hostile sexism toward women was not significantly related to these outcomes or voting in the structural equation models. When accounting for other variables (e.g., Modern Racism), sexism toward women was not a unique determinant.

Of the three factors, U.S. nationalism seemed to play a lesser role. Greater U.S. nationalism was associated with more positive evaluation of Trump, independent of race, age, gender, party affiliation, Modern Racism, and sexism toward women. But, U. S. nationalism was unrelated to voting intentions or behavior. However, the measure of nationalism used in this study assessed general love for the U.S. over other nations. It did not assess White nationalism, which incorporates ideals of placing White interests above other groups’ interests (e.g., [80]). If White nationalism, rather than civic nationalism, accounted for the election results, this may also explain why Modern Racism consistently predicted all of the outcome variables.

The current findings should be taken in light of certain limitations. The data were correlational. Thus, third variables (e.g., anti-immigration attitudes) may explain these findings, and causal interpretations cannot be made. The sample was not representative of the U.S. population. Consequently, the findings may not generalize to the larger electoral body. All measures relied on self-report, so there may be concerns of common method variance exaggerating associations among variables. To address these concerns, future research might examine how implicit measures of Modern Racism, sexism toward women, and U.S. nationalism relate to voting behavior. Further, although standardized beta coefficients can be compared in the SEM analyses, it is important to note that the number of items used to assess each variable differed, which may influence variability in scores. Although Modern Racism was the only factor that predicted voting, that does not necessarily rule out sexism toward women or U.S. nationalism as determinants of actual voting. If the effect sizes of sexism toward women and U.S. nationalism as predictors of voting were small, the sample size of those who voted may have been underpowered. Indeed, for those who returned for the second part of the study and voted, Modern Racism was the only significant predictor of candidate evaluations, voting intentions, and voting behavior (see Tables 6 and 7).

Finally, concerns have been raised about both the construct and measurement of Modern Racism. Specifically, it has been posited that some Modern Racism scales may in fact be measuring conservative beliefs (e.g., everyone should work hard and not receive “handouts;” [67,68]) or beliefs toward political out-groups and immigrants [81,67]. These arguments generally have been levied against the Symbolic Racism Scale, and not the Modern Racism Scale that we used [82,43]. Thus, based on the literature and our data, the concerns regarding measurement of Modern Racism do not seem to pertain to our findings. However, if relevant, these issues may exaggerate the role of Modern Racism.

In conclusion, this study provides empirical evidence for some of the psychological factors that may have been associated with the 2016 U.S. presidential election. Using a prospective design, simultaneous consideration of factors, and a national sample, we found that Modern Racism was consistently associated with 2016 election outcomes. Our findings add to an existing literature suggesting that Modern Racism may help to shape political attitudes and behavior, affecting leadership and policy.
